# Justifiability and AI: putting explainability in its place

**DOI:** 10.1007/s00146-026-03030-9

**Published:** 2026-04-13

**Authors:** Boris Babic, I. Glenn Cohen, Julian Savulescu

**Affiliations:** 1 University of Hong Kong, Hong Kong, China; 2 Harvard Law School, Harvard University, Cambridge, USA; 3 Uehiro Oxford Institute, University of Oxford, Oxford, UK; 4 Centre for Biomedical Ethics, Yong Loo Lin School of Medicine, National University of Singapore, Singapore, Singapore

**Keywords:** Transparency, Explainability, Black box learning, Responsible AI, Ethical AI

## Abstract

As artificial intelligence and machine learning (AI/ML) systems become increasingly pervasive in society, their opacity—i.e., the difficulty, and sometimes impossibility, of understanding why they make the decisions they make—has become a serious problem. This is especially true in sensitive decision-making contexts, such as criminal justice, health care, and finance, or in choices requiring allocation of scarce resources. One attempt to “open up” the AI/ML black box has been the emergence of post hoc explainability algorithms—algorithms which generate post hoc approximations to black box models. However, such algorithms have been criticized as merely providing after the fact rationalizations for the decisions these systems make. In this paper, we defend and articulate a different concept—AI/ML justifiability. We explore several ways in which an algorithm could be justifiable, and we argue that pursuing justifiability is a worthwhile goal. A key to our argument is a distinction from the philosophy of action between motivating and normative reasons: effective explanations require (but are unable to provide) motivating reasons, while effective justifications require (and can indeed provide) normative reasons alone. We conclude that as long as a model is justifiable, it can be trusted even if it cannot be explained.

## Introduction

Transparency in artificial intelligence and machine learning (AI/ML) has become a pervasive problem for scholars, lawmakers, and executives. Many scholars believe that black box AI/ML is problematic from a legal, moral, and policy perspective—we do not want important social choices to be made in a way that makes it impossible to understand why a certain path was taken, or to hold anyone accountable when a mistake occurs (Babic et al. 2021; Wachter et al. 2018; Deeks 2019; Vredenburgh 2022). One attempt to “open up” the AI/ML black box has been the emergence of post hoc explainability algorithms—algorithms which generate after the fact approximations to black box models (e.g., Ribeiro et al. 2016; Lundberg and Lee 2017). However, we have previously criticized such algorithms as being a “fool’s gold” due to the inability of users to discern what is actually happening inside a black box model or to provide meaningful action guidance (Babic and Cohen 2023). Part of our argument against post hoc explainability algorithms rests on a distinction from philosophy of action between motivating and normative reasons. A motivating reason explains an agent’s action—it is the reason, from the agent’s perspective, that moves them to act, regardless of whether it is morally good or bad—while a normative reason justifies, or ratifies, an action—it is a “valid” reason so to speak. We will argue that effective explanations should be able to furnish motivating reasons. And while this is what post hoc algorithms promise, what they deliver is something different—namely, normative reasons (at most).

Meanwhile, the so-called interpretability algorithms (i.e., simple, understandable models) are laudable where they can be developed, but they are difficult to execute with high dimensional models trained on unstructured data, such as text or images (Letham et al. 2015; Angelino et al. 2018; Rudin 2019). The important question then becomes: if post hoc explainability algorithms are not effective, and interpretable models are insufficiently scalable, then what should we do instead? This is a fundamentally important question as the use of large scale general purpose models becomes increasingly pervasive. These models are undoubtedly black boxes, and explaining their inner workings effectively is exceedingly difficult. In this essay, therefore, we defend and try to articulate a different concept—*Justifiability*.^1^

To illustrate, consider a pair of examples:

- **Case 1**: We use a black box AI/ML model to make mortality predictions for individual patients in a health care context (i.e., the model produces an estimate for when a patient will die).
- **Case 2**: We use a black box AI/ML model to make allocation decisions for who will get a limited number of organs available for donation.

Both cases are loosely based on actual algorithms. Case 1 corresponds to a palliative care algorithm developed by researchers at Stanford University for predicting all cause 3–12-month mortality using a deep neural network fit to electronic health record (EHR) data (Avati et al. 2017). Case 2 corresponds to the continuous distribution algorithm for organ allocation presented in Papalexopoulos et al. (2022).

These cases present quite different normative concerns—and explanation is neither necessary nor sufficient for the ethical use of the algorithm in either case. In Case 1, one of the relevant questions would be whether, from the perspective of patient dignity and autonomy, this kind of information is something we should provide to them. Simply explaining to the patient how the mortality prediction was made would not alleviate any concerns about the disruptive effect that such information might have on the course of their life. In Case 2, one of the most relevant questions would be whether the ultimate pattern of distribution of organs is normatively satisfactory. And to answer that question we need to provide morally persuasive reasons—i.e., a justification. Explainability is, again, neither necessary nor sufficient.

In this essay, we articulate several ways in which an algorithm could be justifiable, and we argue that pursuing justifiability is a worthwhile policy goal across a wide variety of decision-making contexts. We argue that one promising account is that justifiability is constituted by a sufficiently acceptable ethical justification for the distribution of expected benefits and harms which result from the human use of AI/ML. We explore several strategies for sufficiently acceptable ethical justification. We propose that as long as a model is reliable and justifiable, it can be trusted even if it cannot be readily understood. While we chart the conceptual space of justifiability as much as possible, we leave some open texture for further research with respect to how exactly justifications should look, including what kinds of reasons should be provided, how to balance competing moral theories, and whether justifications should ever be automated.^2^

The essay proceeds as follows. In Sect. 2, we discuss interpretability and explainability and we argue that explainability fails to underwrite the moral burden of automated decisions – it is neither necessary nor sufficient for trust, and explaining an algorithmic decision does not in general make it more just or ethical. In Sect. 3, we develop the general notion of justification carefully. We focus on its relationship to trust and understanding (Sect. 3.1); we compare it to legal decision-making (Sect. 3.2); we distinguish between normative and motivating reasons and we argue that algorithmic decisions should be grounded on proper normative reasons (Sects. 3.3–3.4); and we consider various facets of justification in practice, including how it can be operationalized (Sect. 3.5). Section 4 concludes.

## Interpretations and post hoc explanations

Critical decisions in finance (e.g., Capponi and Lahalle 2023), medicine (e.g., May 2021), and public policy (e.g., Karadeglija 2024) are increasingly made by large scale artificial intelligence and machine learning (AI/ML) systems. Often, these systems are black box decision-making models, because their underlying architecture or the sheer size of their dimensionality makes their predictions difficult to understand by human users.^3^ This is especially true with general purpose large language and foundation models, which are multi modal, containing both supervised and unsupervised components, and trained on datasets involving trillions of parameters.

As the pervasiveness of these systems increases, so too do the calls to keep them accountable and understand how their decisions are made. There is an increasing push by policymakers to make AI/ML systems transparent, and some scholars have even argued that the EU General Data Protection Regulation (2016/679) contains a right to explanation for algorithmically generated decisions (Selbst and Powles 2017). Likewise, there are draft rules in other countries which would require explanations of AI/ML predictions. For example, the Canada Bill C-27, also known as the Digital Charter Implementation Act, 2022, would require the system owner to produce the “reasons or principal factors that led to the prediction” (Bill C-27, 2022). For modern large language models such as ChatGPT-4, or large image models such as Dall-E2, it is far from clear that producing the “reasons or principal factors” is at all possible. In this section we explain why that is the case and, more generally, we will carefully describe two different families of approaches for increasing the transparency of an AI/ML system, namely, interpretability and (post hoc) explainability (Babic and Cohen 2023).

### Interpretability

The interpretability paradigm is intuitive and easy to grasp. In short, the idea is simply to replace a complex model which is impossible to understand with a very simple model which is easy to understand (Rudin 2019). But this is not as easy as it seems.

One might wonder why we do not always do this, if it really is that easy. Part of the reason is that given a complex model, it can be very difficult to identify an equally well performing sufficiently simple model. And in some instances, it is possible that such a model does not even exist (Dziugaite et al. 2020). The latter concern is particularly pressing in the world of large foundation models. One clue as to whether we can find a simple model that performs as well as a very complex one is whether there is a sufficiently low-dimensional representation of the high dimensional data that the complex model is trained on. Often there is, but finding a sufficiently low-dimensional representation can be challenging (See e.g., Buchanan et al. 2025).

### *(*Post hoc*) Explainability*

Meanwhile, in the post hoc explainability paradigm, the path to explanations is very different.

#### The LIME algorithm

Consider one of the leading such algorithms, known as LIME (Ribeiro et al. 2016) (we consider another leading explainability algorithm, known as SHAP, below). The LIME algorithm takes a black box model used for estimating a classification boundary (for example, in order to determine which applicants receive a loan and which do not), and it also takes a particular individual for whom a prediction was made (say Steve, an applicant who was denied a loan). The algorithm then constructs a surrogate linear model after the fact (this is why it is post hoc) which is such that had that linear model been applied to the case of Steve, then on that linear model Steve would also be denied the loan. Yet it is not clear how this constitutes an explanation.

Pursuant to post hoc explainability approaches, the explanation is typically assumed to consist in the parameters of the linear model (for example, these may correspond to Steve’s income or his debt levels). The assumption is that all linear models are transparent and that we can explain black box models by approximating them with linear ones—and then reporting to the person requesting an explanation a story that is based on the parameter coefficients of the linear model (e.g., had your income been *x*% higher, you would have received a loan).

There are many problems with this approach, some of which we discuss further below (see also Rudin 2019; Babic et al. 2021; Babic and Cohen 2023), but the most salient is that this is a rationalization of the decision-making process for a particular individual: the parameter coefficients of the linear model given to Steve would not be the same if they were given to another individual, say Stacey, because a linear classification boundary cannot be globally faithful to a non-linear one (Dasgupta et al. 2022). The reason for this is geometrically very simple: a straight line can be approximately tangent to a curve at a particular point, but it cannot approximate it well everywhere.

#### The SHAP algorithm

Another leading explainability algorithm, SHAP (Lundberg and Lee 2017), is conceptually similar in the sense that it is a post hoc additive feature attribution method, meaning that the parameter coefficients and their associated input variables can be added together to compute the prediction. The goal is again to use a simpler model after the fact in order to explain the original black box model. But the way that SHAP accomplishes this is a little bit different from LIME. Shapley values originate from cooperative game theory, where the goal was to quantify a player’s marginal contribution in a collaborative game (for example, an author’s relative contribution to a multi-authored paper or a friend’s relative contribution to a joint bill) (Shapley 1951).^4^ This method became useful to statisticians, because it could equally well be applied to quantify a variable’s relative contribution to a model, which is particularly useful under conditions of multicollinearity. To extend the model to this context we simply think about the predictors as the players in this “game” and we think about some performance metric of the model (such as the cumulative proportion of variance explained) as the payoff or gain within that game. This came to be known as Shapley value regression (Lipovetsky and Conklin 2001). Lundberg and Lee (2017) take this approach, and apply it toward generating post hoc explanations by identifying a procedure for approximating Shapley values for each feature from any model. In general, our comments about LIME apply equally to SHAP and vice versa.

LIME and SHAP have a few things in common: they are both local post hoc approximations of one way the features could be added to produce a prediction that was in fact observed. But they are not laying bare the internal architecture of the model, nor can they approximate any model everywhere.^5^

## Justification

It is assumed in the literature on Explainable AI/ML that trust requires understanding – hence the felt need to “open up” the AI/ML black box. However, as two of us have argued previously, explainability methods, as described above, do not in general contribute significantly to our understanding of how a prediction model works (Babic and Cohen 2023). The simple reason is that they are post hoc surrogate models used to rationalize a particular prediction that is made by a black box model. This is bad news for post hoc explainability algorithms, but it is not bad news for building trustworthy AI/ML systems. Fortunately for us, it is possible for an agent to have trust in a system without that agent (the trustor) understanding how it works (see also Ahn et al. 2024).

### Trust without understanding

While understanding can lead to trust—a complete causal explanation of a process could be sufficient to trust it—it is not necessary for trust. There are many processes where we observe trust in the absence of understanding. For example, in medical discovery it is not unusual for a therapy to be identified, marketed, and widely used before we have a complete understanding of how it actually works. And in many cases, such a complete understanding never arrives. For example, acetaminophen/paracetamol effectively alleviates pain. It is widely accepted, used, and trusted, and has been for decades. Yet we still do not fully understand how it works and recent research continues to shed further insight on its therapeutic pathways (Toussaint et al. 2010).

The reason we trust such discoveries has less to do with our causal understanding of the mechanism by which they operate, and more to do with the social/institutional infrastructure within which they are developed, brought to market, and used (London 2019). For instance, when a drug goes through extensive clinical testing and large, multinational and multiphase clinical trials, this lends credence to its safety and effectiveness. The legitimacy and credibility of the certifying institution matters as well, and so do the underlying values that the institution protects. For example, an FDA approved drug would not be trusted if there was a general belief by the public at large that the FDA has no interest in protecting patient safety. Hence, our trust in a product or system once it is brought to market is relatively independent of our technical understanding of how it works. It is instead mediated through the social/institutional factors within which we interact with it – in a health care context, it depends on the legitimacy of the underlying public health agency, the participating hospital networks, public health messaging, and our own physician’s views, to name a few.^6^

Furthermore, understanding is not a sufficient condition for trust either. For example, imagine we have a very effective, simple and transparent algorithm designed to produce fair allocation outcomes for managing organ donations queues. This algorithm relies on legitimate and socially accepted factors and it weighs them appropriately. Except for one wrinkle, if the patient drives a red sports car, their likelihood of fair allocation is reduced by 1/10. The point of this illustration is to add an element of randomness to an otherwise fair and transparent process. Would understanding of this algorithm improve trust in it? Certainly not—it would actively diminish it.^7^ When one learns about the arbitrary element contained in the algorithm, one would no longer trust it, despite its perfect transparency. In short, then, understanding is neither necessary nor sufficient for trust.

This is in line with the argument in Robbins (2019). Robbins argues that explanations should not be required of the entities making a decision—whether that be a doctor or an algorithmic system—but rather of the decision itself. We are sympathetic to this line of reasoning, and one can see the argument we give here as an attempt to provide a way of doing what Robbins suggests—i.e., justifiability is precisely a way of explicating the decision itself, instead of the decision-making entity.^8^

### Legal decision-making

The preceding example of acetaminophen/paracetamol illustrates that its use is justifiable, even though we do not fully understand how it works. Perhaps a better analogy, however, can be drawn from the context of legal decision-making. Consider the case of an appeals court judge reviewing a trial decision. In American federal courts, in many forms of appellate review the appeals (circuit) court will not typically revisit or relitigate the facts of a case as determined by the trial court, meaning that it will treat the factual record as “frozen” on appeal. The appellate judge’s role is typically to evaluate the trial record and determine whether, taking the facts as determined by the trial court, there were sufficient legal errors that merit reversing the decision or remanding the case for retrial.

Importantly, it is widely understood that when the appellate court judge reviews the determinations of the trial judge on issues like the credibility of a witness, he or she should not attempt to substitute the trial judge’s opinion with her own. Nor does he or she try to understand the true motivations of the trial judge—he or she only seeks to understand whether errors have been made and if the articulated reasons are sound. This is sensible procedurally—as we do not want the appellate process to be unduly repetitive. But it is also a recognition of the open texture of legal facts: it is understood in jurisprudence that there can be multiple legal pathways to the same decision, and hence, multiple opinions can lead to the same outcome (Dworkin 1986).^9^ Similarly, we understand from insights in behavioral decision-making that a judge’s true causal reasons for a decision may not be readily apparent from her opinion (Danziger et al. 2011; Tversky and Kahneman 1974).^10^ Accordingly, when an appellate judge reviews a trial judge decision, they accept the explanation that is given in the opinion and they review that decision as given. It does not matter, legally speaking, if they also believe the trial court judge was motivated by something different than the explanation given. For example, that the trial judge as a former prosecutor is overly favorable to police and prosecutors. They review the quality of the reasons given, and not what actually may have motivated it. In other words, they do not try to open the black box of what actually drove the judge to their choice.^11^ The general point, however, is that what matters is not so much why a judge made her decision, but whether that decision is justifiable and indeed justified. Furthermore, the true causal reasons may be inaccessible both to outsiders and to the judge. More generally, the true reasons for why we act are not readily transparent to us, and indeed the human mind is much like a black box, notwithstanding our advancements in understanding the neural architecture of the brain (Bonezzi et al. 2022).^12^

### Motivating reasons vs. normative reasons

In our legal analogy, the appellate court judge does not attempt to open up the black box, so to speak, of the trial judge and to peek into her mind. Rather, the appellate court judge asks whether the decision is justifiable. To borrow a distinction from philosophical ethics—which will be important to our argument—we can distinguish between motivating and normative reasons (see Dancy (2000) for an overview). Motivating reasons are the reasons that move us to act—that bring about or guide an action. We could think about these reasons as causes (however, nothing in our argument will turn on whether one accepts that reasons can be causes). Meanwhile, normative reasons show why an action is permissible, ethical or optimal, by reference to some normative standard (Radcliffe 2008). As Scanlon (1998) famously puts it, a normative reason is “a consideration that counts in favour of” an action (p. 17). Similarly, to paraphrase Parfit and Broome (1997), when we ask what action is most rational for someone to take, we are asking about normative reasons. And when “we act for that reason, it becomes our motivating reason” (pg. 99).

Normative reasons, for Parfit, are objective and external, while motivating reasons are bound up with the agent’s mental states (how exactly they are bound up is a matter of great disagreement, but that can be set aside for the purpose of our argument in this essay). The theory of justifiability that we will propose is a theory which focuses on identifying normative reasons, because (we will argue) those reasons are relevant to the moral assessment of an AI system’s actions. Dancy (2000) provides a helpful summary of the basic distinction:

“When I call a reason ‘motivating’, all that I am doing is issuing a reminder that the focus of our attention is on matters of motivation, for the moment. When I call it ‘normative’, again all that I am doing is stressing that we are currently thinking about whether it is a good reason, one that favours acting in the way proposed (pp. 2-3).

Consider an example. John believes that he may have caught a blood borne contagious disease. The blood donation van makes its monthly visit to his university. He decides to donate blood for the first time. His reason is that he wants to know if he has the disease, knowing that the blood transfusion service will test his blood for this disease and it will notify him if he is carrying the disease. He knows that he could go to his doctor for a blood test, but he decides that he cannot be bothered and this will be easier. Did John have good reason to act as he did? From the moral perspective, should he have acted as he did?

A reason for acting is a fact or circumstance forming a sufficient motive to lead a person to act. Knowing a person’s reasons allows us to understand why a person acted as he did. John’s reason for donating blood was a desire to find out whether he had a certain disease together with the belief that donating blood was the best way to achieve this goal. This reason explains why he acted as he did. It has been called an explanatory or motivating reason.

Good reasons for action are normative or justifying reasons for action. A reason for action is good if it meets a standard, that is, if it conforms to a set of norms governing that behavior. In one sense, John had a good reason to act as he did: if his beliefs were true, donating blood would be an effective way of finding out if he has the disease. However, John could equally effectively have resolved this uncertainty by having a test performed by his doctor. This course of action is better in another way. It eliminates the possibility of placing others at risk by using blood donation as a means of testing. John knows this fact. Therefore, John does not have good normative or justifying reasons for acting as he did, even though he has good motivating reasons for acting as he did. And on balance, we might conclude that John has most reason not to donate blood but to go to his doctor for a test first.

In a similar way, if we return to the judicial context, consider a judge who has to interpret a statute before applying it to adjudicate a particular case. For the sake of the example, let us say the judge has to decide whether the phrase “navigable waters,” in the US Clean Water Act, includes wetlands or not. The judge’s interpretation of the statute may be persuasive, or unpersuasive, in light of the reasons given in support of it. For example, are American wetlands in general connected to rivers? How many wetlands have historically played a role in maritime navigation? Is there currently a demand for navigation through wetlands? What kinds of evidence can we adduce in support of the drafters’ intention to include or exclude wetlands? Etc. Importantly, the judge’s subjective motivation (in our example, whether or not the judge wants to protect wetlands or not) is irrelevant to the persuasiveness of the proffered interpretation. The reasons given to support the interpretation are in general independent of the judge’s psychological motives underlying the decision.

The core conceptual difference between the theory of justifiability that we attempt to sketch here and the post hoc explainability approaches that we have criticized is that justifiability is fundamentally a notion that involves articulating normative reasons for an algorithmic decision, whereas post hoc explainability attempts to articulate motivating reasons (and ordinarily fails). This is the crux of our argument against post hoc explainability as a form of transparency, and as a means to shoring up trust—it is not that we are against it, it is that we think it is doomed to fail. As we have argued (Babic and Cohen 2023), this can have further detrimental consequences. When people are presented with an explanation, which is later revealed not to be faithful, reliable or stable, this leads to further disillusionment with that decision-making system.^13^ Meanwhile the notion of justifiability that we develop does not promise motivating reasons. It promised only what it can deliver—namely, normative reasons.

### AI predictions and normative reasons

Consider now an example of algorithmic decision-making. Suppose that we use an automated system in order to determine who gets a kidney donation in a context where there are not enough organs to distribute to everyone in a queue who needs a transplant. Suppose further we have a particular patient (let’s call him Tom), and Tom has been placed fairly low in the queue by the algorithmic decision-making system. Tom demands an explanation or justification for why he was placed so low in the queue.

As we have argued, and as others have argued, a post hoc explanation of the form produced by explainability algorithms such as LIME (Ribeiro et al. 2016) or SHAP (Lundberg and Lee 2017) is not a normatively valuable reason for the decision to present to Tom. The explainability paradigm promises to offer a motivating reason. And in light of that promise, Tom expected a motivating reason. However, the method then fails to produce what he expects—because the post hoc explanation is not a motivating reason. At the same time, the post hoc explanation does not offer a good normative reason either, because it searches for an explanation through post hoc model approximation—an automated and technical process which does not reliably track moral considerations. In a similar fashion, we could describe the limitations of the automated post hoc explainability algorithm by drawing on Dennett’s so-called “intentional stance” (Dennett 1987). Intentionality, for Dennett, requires certain folk-psychological assumptions about the agent whose behavior is to be explained—namely, that they have beliefs, that they have desires, and that they act rationally in a way that maximizes the expected value of those desires in light of their beliefs (Dennett 1971). Nothing of this sort can emerge from mechanistic post hoc explanations (Note, however, our criticisms of post hoc explainability do not *require* any reference to intentionality or folk-psychological attitudes being attributed to machines). But so then, what would be an adequate normative justification?

First, in order to provide an adequate justification, we must confront the fact that we are in general operating in the world of black box models. In the simple case, where a very easy to understand model is being used for making predictions (for example: using a person’s age, weight and BMI to predict their risk for diabetes) justifiability and explainability are less of a concern. But in any high dimensional models, including computer vision, genetic data, or electronic health records (EHR) data, the model will be too complex to be interpreted. In these cases, motivating reasons for action (even if they are desirable) are not accessible to us. Hence, providing adequate justifications requires engaging in moral reasoning. Whether or not Tom’s decision is justifiable depends on whether it comports with our moral commitments as a society.

For example, we would first look at the expected costs and benefits of placing Tom in a particular position on the queue relative to other patients. To assess the benefits, we should evaluate the extent to which Tom needs a kidney, the likelihood of transplant success, and his overall ability to cope with major surgery. We may also want to compare Tom’s age and expected survival rate, or, his expected quality adjusted life years (QALYs) against those of other patients. Such considerations are utilitarian. This is not the only form of acceptable moral reasoning. We may have Kantian or deontological intuitions as well. For instance, we may hold that Tom’s race, ethnicity, or religion should never enter into the decision-making calculus, and we may wish to hang onto this commitment regardless of its consequences. This is just an illustration of the kind of reasoning process that may enter into a decision of where to place a patient in an organ donation queue—but it should be clear that this kind of justificatory process is fundamentally different from the automated and technical process of producing post hoc explanations. Indeed, the United Network for Organ Sharing (UNOS) administers the Organ Procurement and Transplantation Network in the United States in a way that attempts to strike a balance between competing moral considerations, such as the ones described above in the process of justification.

Now, to illustrate how a decision may or may not be justified, consider an extreme case: there are 100 patients in the queue, 50 of them are white, and 50 of them are black. All the Black patients are between 21 and 30 years old, and all the White patients are between 51 and 60 years old. All the Black patients are in good health, and all the White patients are in poor health. Our decision-making algorithm has produced the following outcome: positions 1–50 are all occupied by the White patients, and positions 51–100 are all occupied by the Black patients. Tom is a Black patient and occupies position 57. While there can be more than one way to justify a decision—justifications are typically not unique—it is hard to see in this extreme case how Tom’s outcome could be justifiable.

One could try to justify it, though. For instance, it may be worth distinguishing between a commitment to a procedurally neutral system and a commitment to a particular distribution of outcomes. If justifiability is about procedural justice—in the former sense—and not about the distribution of outcomes—then perhaps we can envision situations where Tom’s outcome was justifiable. For example, imagine a situation where the actual process was random – queue spots were determined by a coin flip, and each person’s relative position was decided through the same random mechanism. One could argue that the outcome is justifiable regardless of its racially unequal distributional consequences. This raises a question: what are we willing to consider a sufficiently acceptable ethical justification for a decision-making process? Should we look at the process’s commitment to certain procedural norms (such as neutrality in decision-making) or should we look at the distribution of outcomes? Or both? We do not ultimately take a position on this as we suspect the answer will be context and industry sensitive. For example, using a random process to urgently distribute vaccines in short supply could actually be justifiable in a medical emergency. Meanwhile, using a random process to decide which of several patients in an organ donation queue ultimately get an organ in limited supply seems like a morally wrong way to justify the distribution of scarce resources in ordinary circumstances. In short, justifiability is constituted by a sufficiently acceptable ethical justification for the distribution of expected benefits and harms. This justification can come from an analysis of the process that led to the outcomes, or an analysis of the outcomes themselves, or both. The central point is that it is a right to a justification, not a right to an explanation, that really matters to the subjects of automated decisions. This applies to all algorithmic decision-making, whether it be automating hiring, allocation of social care, or the granting of loans.

### Facets of AI justifications

In this section, we discuss several important facets of justification that arise as we consider how to operationalize this concept.

#### *Ex ante or *post hoc*?*

*First*, justifications, like explanations, may be produced post hoc—they are often developed after a particular decision has been made. However, since they attempt to provide normative reasons, and not motivating reasons, the post hoc nature of justifications is not necessarily problematic. Justifications are analogous to appellate review in this case. For example, imagine a simple case where we have one criterion for who to treat in the context of a flu outbreak—namely, the sickest patients, as measured by some objective indicator like fever, lung capacity/risk of pneumonia, or the like. In this situation, we would indeed want to examine the distribution of outcomes to see whether that criterion has been satisfied: have we indeed treated the sickest patients? Have we applied the best measure of sickness? But our analysis of distributional consequences does not have to stop here. For example, we may also notice that our commitment to treat the sickest patients has led to a severe racial imbalance—for example, we learn that under this guideline, the probability that a Black patient is refused treatment is four times higher than the probability that a White patient is refused treatment. This could happen because White patients are overall sicker (which would not undermine the normative force of our rationale) but it could also happen because, for example, the Black patients’ sickness is more difficult to identify leading to more false negatives (which may well undermine the normative force of our rationale). Depending on why the racial imbalance occurs, we may find our initial commitment to treating the sickest patients problematic. This case demonstrates how we must consider whether our justification—i.e., the commitment to treat the sickest patients—remains compelling in light of the societal outcomes it has produced (i.e., racially uneven distribution). Viewed in this light, our notion of justification shares a family resemblance to Smart and Kasirzadeh (2025)’s notion of a socio-structural explanation. We both focus on aspects of an algorithmic decision that go beyond post hoc explainability algorithms. Some scholars distinguish between mechanistic and non-mechanistic interpretability, where mechanistic approaches attempt to reveal a system’s underlying architecture, while non-mechanistic approaches focus on illuminating the estimated function connecting inputs to outputs. The latter is impossible, as Arvan (2025) argues, and the former is misleading, as Babic and Cohen (2023) argue. However, justifiability is different from both such notions. Like Smart and Kasirzadeh (2025), we focus on the social concomitants of algorithmic decisions, but whereas they draw on one kind of ethical approach to underwrite their argument (Iris Marion Young’s Social Connection Model (Young 2006)), we veer more closely toward a different and more consequentialist framework for applied ethics problems (cf. Daniels 2007). There is a meaningful difference between these two. For example, Young’s social connection model attempts to articulate a notion of responsibility for wrongdoing without blame—which is particularly important in cases of complicity for wrongdoing or where a person’s connection to a wrong is remote. But we do not shy away from blame: if an algorithmic decision is indeed unjustified, then the people responsible for that decision are indeed blameworthy.

#### Justifiability of what?

*Second*, as we alluded above, when we discuss justifiability, we can further ask: justifiability of what? We can focus on justifying a certain distribution of outcomes at a particular point in time. Or, we can instead focus on justifying a decision-making process over time. To illustrate the difference, consider a followup to our previous example: imagine after learning that our commitment to treat the sickest patients has led to a racially imbalanced distribution in who we decide to treat, we suddenly drop that commitment and decide instead to treat the poorest patients. This reverses the distribution with the consequence that the new batch of treated patients is predominantly black. From a static perspective, if we look at the full group of treated patients, we may well find that it is relatively racially balanced (assuming equal numbers and prevalence rates). However, the process may still seem objectionable: we switched rationale mid-way from treating the sickest to treating the poorest. At the very least, it would seem we ought to be transparent about the fact that we made a switch and the reason why we made a switch, and that reason might itself require justification. When proffering justifications, therefore, it will be important for decision makers to consider how to appropriately strike a balance between a commitment to a certain process and a commitment to certain outcomes.

#### To automate or not?

*Third*, justifications are not automated. Post hoc explanations are automated by algorithms like LIME (Ribeiro et al. 2016) or SHAP (Lundberg and Lee 2017). But we take justifiability to be a human centric concept with moral reasoning at its core. It is worth considering whether justifiability could be automated, and there is some emerging research on automating moral reasoning. However, at a first pass, we are skeptical of attempts to do so. The descriptive facts that we can collect and train a model on cannot determine which ethical principle we should adopt or prioritize. The normative decision to decide right from wrong is arguably inherently human and cannot (and should not) be outsourced to a machine, at least at present. Another way to put it is that we cannot derive values from facts. Machines are good at retrieving facts, but not so good at adjudicating competing values.

Many philosophers are metaethical moral realists (e.g., Railton 1986), which means that they think about moral facts in the same way that we understand mathematical facts—objective truths of the universe which can be discovered (Clarke-Doane 2012, 2014). However, we have no “moral microscope” with which we can observe moral facts. In practice, moral facts are often reduced to what the majority believes is right—and this can be problematic. While AI/ML may be able to figure out what the majority believes is right, we are doubtful that such information answers the question of what is actually right. For example, this kind of assumption—the assumption that we can crowdsource morality, so to speak—is at the very core of the now well-known Moral Machine project (Awad et al. 2018). In that context, researchers have participants play a trolley style game, where the game involves making decisions about who should be saved, and who should be injured, by a self-driving car. The researchers then attempt to draw moral conclusions about right and wrong according to the most frequent preferences people hold, for example, saving more rather than fewer lives. But we are skeptical of this notion of morality by majority rule. Indeed, one of us has argued that using data about public preferences requires a procedure of reflective equilibrium—a deliberative method of moral reasoning popularized by Rawls (1971) whereby we adjust our beliefs until we reach a socially stable point—that brings moral theories, principles, and concepts into maximum coherence with public intuition (Savulescu et al. 2021, 2019).

Moreover, there are some obviously intuitive examples of moral facts that remain true regardless of how many people disagree with them. For instance, even though we can say that slavery in America can be understood in its context, it remains the case that it was as morally wrong then as it is now. And that is true regardless of how many people supported that institution during its existence. In other words, it is possible for a population to have unanimous agreement about a practice (such as slavery) and for that practice to still be morally wrong. Hence, the idea of crowdsourcing morality, which is presupposed by the practice of automating morality using only public preferences, is misguided.

#### A right to justification?

*Fourth*, it is an open question when we should have a duty to produce justifications. For some high frequency prediction systems (for example algorithmic trading in finance), it would not be sensible to expect justifications of individual predictions. For very sensitive individual decisions involving distributions of scarce resources (such as medical care or government contracts), justifications seem particularly important—it is something the individual might make a rights-based claim to have provided. And for other decisions between these two extremes, we may want to design a system analogous to appellate review—where it is possible to apply for a justification, but a justification is not provided as a matter of course. This is consistent with emerging work on appellate systems for AI/ML decisions (Cohen et al. 2023). However, the comparison between appellate review and seeking a justification is not perfect—since appellate review is a check or a “second look” at an original justification which is different from one’s claim to have a justification in the first place.

It is worth considering why some contexts seem to require justifications more than others. This question of *when* to require justifications in the first place would have to be answered, from the perspective of a social policy designer or a community, before we can get to the main question of *how* to provide justifications or which kinds of justifications are compelling. Our intuition above is that sensitive decisions and decisions involving scarce resources are particularly compelling candidates for mandatory justifications. Other candidate contexts where we likely need justification would be automated decisions which substantially affect the allocation of burdens and benefits (for example, automated legal judgments) or decisions with very severe consequences, such as when AI systems are deployed on the battlefield. Why might that be? We can think of a few reasons for this, though we do not claim they are exhaustive.

First, as a check on power. While false justifications can be generated, requiring justifications forces the decision maker to say something about the decision that was reached, and that something may then be subject to scrutiny. Consider our previous example: if the decision maker says we treated the poorest patients in the case of an influenza outbreak, we can ask: was that the right thing to do? There are analogous contexts in law where justifications are used as a check on power. For example, placing the burden of proof on the prosecution in criminal trials forces the state to say something about why the defendant is charged with the particular crime and whether that crime’s elements are met—and that something can be challenged on a subsequent review. We also require this in pseudo-formal proceedings. For example, we may require a grant reviewer to write the reasons for her decision.

Second, in human decision-making it may be that the act of justifying a decision can improve the quality of decisions overall because it provides the decision maker with a “stop and think” moment. This consideration is particularly interesting to think about in the context of AI/ML. Does the AI/ML need anything like a “stop and think” moment? Perhaps not, however, a better way to think about the value of the justification is that it provides us as a society with a “stop and think” moment. If treating the sickest patients leads to wide racial disparity, then we may want to reconsider why that is occurring and whether it undermines the normative force of our criterion.

Third, one may consider the speech act value of a justification—its effect on affirming the dignity and autonomy of the person who is adversely affected by a decision. In philosophy of language, a speech act is an act that can be performed by the proposition being expressed (e.g., “I quit”).^14^ If a patient is refused care, we may think that we *owe* them a morally sensible story for why they were refused care. Even if that justification does not improve the quality of the reasoning, check power, or provide an actionable form of recourse, it may nonetheless be a way of respecting individuals and acknowledging that they may end up not having their needs met.^15^

Fourth, there are also some less palatable rationales of justification. One may see justifications as nothing more than rationales for a particular distribution of outcomes—as a sort of opiate for the masses whose goal is to placate, suppress criticism, and delude the recipient into acceptance. Naturally, we do not think this is a good justification for a justification, and if that is the best defense of having it in a certain context, we may wish to reconsider.

It is a strange quirk of the sociology of AI/ML ethics that explainability has come to be understood as an attempt to identify motivating/causal reasons for action or to open up the black box. Part of the reason for this may be that we have imposed human standards on machines: we want to know people’s motivations so we can decide whether to trust them in future interactions. Hence, we want to know a machine’s motivations so we can decide whether to trust them in future interactions too. But machines do not in general have readily discernible motivations; they are only useful or not useful. And even in human interactions, mechanistic explanations are not always the sort of thing we are after.

For example, if we think about what kinds of explanations have been sought in the courtroom or in medical care, they are generally of the form that we are calling justifications. And even in everyday moral practices, we seek justifications, as opposed to mechanistic explanations. For example, if an appellate judge wrote an opinion in which she tried to describe all the reasons that moved a trial judge to act, down to the last detail, we would not find that particularly valuable. Indeed, providing all the reasons for action down to the last detail may well be corrosive to the rule of law as an institution—because it risks losing the forest for the trees. Similarly, if a child misbehaves and, let’s say, throws a rock at a window, and the parent asks the child to explain themselves, the kind of explanation that is sought is one that provides normative reasons, as opposed to the literal reasons that motivated the act. That is to say, if the child begins by describing “I moved my left arm to pick up the rock, then I bent over to reach it, then I assumed I could throw it as far as the window…” we would stop the child and clarify that we want a justification, and not a mechanistic explanation: “why did you think that throwing the rock at the window was acceptable?” So it appears that we have known all along what counts as a good explanation in our moral and legal theorizing,^16^ but somehow we went off track in the post hoc explainability literature, and in that sense this article is an attempt to bring us back on track.

## Concluding remarks

The aim of this project is to shift the discussion on AI/ML decision-making away from mechanistic explainability and toward substantive moral reasoning in the form of providing ethically compelling justifications for AI/ML decisions. We have argued that mechanistic explanations are neither necessary, nor desirable, for ethical AI/ML decision-making. And we have argued that we should instead focus on justifying automated decisions and the outcomes they produce. This project is more of an attempt to open up that discussion than it is the final word on the subject. Finally, we have explored how such justifications might look and compared them to other contexts in law and health care where similar justifications play an important role. In short, justifications of algorithmic decisions should seek to produce normative, not motivating reasons; justifications are (in general) produced after the fact; justifications should engage in appropriate moral reasoning (including an analysis of both outcomes/decisions made by algorithmic systems and the process by which the outcomes/decisions are generated); justifications should in general not be automated; and justifications should be transparent and accessible.

## Data Availability

No datasets were generated or analysed during the current study.

## Footnotes

1 This concept has a long history in both pure philosophy—i.e., justification of belief (Gettier 1963)—and in political philosophy—i.e., in justificatory reasons (Gaus 1990). Recently, there has been increasing interest in applying the notion of justification to the governance of AI/ML models (e.g., Hadfield 2022 and Loi et al. 2021). For Hadfield, justifiable AI is intertwined with judgment and our normative systems, but given that her piece is a short blog post, understandably it provides limited detail about how exactly justifiability relates to these notions. Meanwhile, Loi et al. focus on what they call the design publicity of an algorithmic system.

2 Indeed, there is already some emerging research exploring how to operationalize justifications in the service of creating more trustworthy AI/ML models. For example, see, Zhou and Joachims (2023), Henin and Le Métayer (2022), Kolářová and Schmude (2025), and de Fine Licht and de Fine Licht (2020).

3 Sometimes the systems are black boxes simply because they are claimed as a trade secret by their developers (Rudin 2019).

4 To understand how a Shapley Value quantifies an individual’s marginal contribution in a collaborative game, consider the following toy example: Alice and Bob plan to take a ride hailing service together. Alice has a better rating, hence, if Alice calls first it costs 10 dollars, and when Bob joins it costs another 10 (total 20 dollars). But if Bob calls first it costs 15 dollars, and when Alice joins it costs another 5 dollars (total 20 dollars). Alice’s fair marginal contribution to the car fare (her Shapley Value) is (10 + 5)/2 = 7.5 dollars, while Bob’s is (10 + 15)/2 = 12.5 dollars, and on this basis we could suggest that Alice should contribute 7.5 dollars and Bob should contribute 12.5 dollars to the 20 dollar fare. While this split is not equal, it is still better for both Alice (7.5 < 10) and Bob (12.5 < 15) to share the car than to ride alone. There is also an underlying rationale for the inequality—for otherwise, Bob would freeride on Alice’s better rating.

5 For additional critical commentary casting doubt on the usefulness of post-hoc explanations, see e.g., Balagopalan et al. (2022), Bove et al. (2024), Freiesleben and König (2023), and Rudin et al. (2024).

6 We do not mean to suggest that situating trust in institutions is an unalloyed good—it certainly comes with risks; for example, institutions may abuse such trust and their legitimacy and credibility may be exaggerated. Rather, we wish to highlight that when it comes to engendering trust, institutional factors often (perhaps ordinarily) overshadow the role of technical understanding.

7 Note, however, that either understanding or lack of understanding can indeed be a sufficient condition for distrust. For example, someone could be skeptical of an algorithm because it is a black box to them; and similarly, someone could lose trust in an algorithm when they learn that the process it uses is unreliable (our own example is an instance of this).

8 Though one area where we disagree with Robbins (2019) is his Catch 22 argument, namely, the claim that “if we have already figured out the acceptable considerations for making the decision … there is no need to use ML in the first place” (pg. 512). If this were true it would actually be good for us in further reinforcing the need for justifiability above all else. But it is not quite true, because it is possible to know all the acceptable considerations for a decision without knowing what their relative contribution to the decision should be – and that is something we can still use AI/ML systems for. For example, we could agree to that the only two considerations for admitting students to law school should be their college GPA and their LSAT score, without knowing what is the best combination of LSAT and GPA for admitting the most talented students.

9 To be clear, we do not take the extreme legal realist view that legal decision-making is purely subjective or political; rather we only presuppose that the legal path to a particular decision is often not unique. See e.g., Kozinski (1993).

10 We use the expression “causal reason” to describe the actual reason but for which the agent’s intentional action would not have occurred. Our usage of this concept is consistent with Davidson’s account of action (Davidson 1963), and in the next section we will draw a more precise definition between motivating reasons (which are causal on our account) and normative reasons (which are not).

11 It is worth recognizing that one can find extreme exceptions to our general illustration—of course, if an appellate judge were presented with evidence that the trial judge made her decision by accepting a bribe, that would be relevant.

12 Kahneman et al. (2021) refer to unexplained variability in human judgments as noise. While noise can be managed, and sometimes brought to a minimum, it can rarely be eliminated. This further reinforces the need for a normative justification of judgments—be they human or machine.

13 By comparison, interpretability approaches which substitute a black-box decision-making system with a simple one are not vulnerable to this criticism. Indeed, we remain in favor of direct interpretability approaches, such as using linear models or decision trees in critical decision-making tasks (e.g., Letham et al. 2015; Angelino et al. 2018). The main problem with these approaches is their scalability: ordinarily, developers or leading frontier models do not want to stop applying those models and replacing them with simpler ones. And in the context of unstructured high-dimensional data, doing so may not even be possible.

14 Austin (1975).

15 Narratives of course can be misleading – and to the extent that a proffered narrative is misleading, it is to that extent a worse justification.

16 See generally, Miller (2019).
